# Relative Brain Perfusion Differences in a Patient With Persistent Cognitive Symptoms: A Case Report

**DOI:** 10.7759/cureus.115893

**Published:** 2026-09-07

**Authors:** Binh Pham, Georgi Stoychev

**Affiliations:** 1 Sleep Medicine, Amen Clinics, Costa Mesa, USA; 2 Integrative Medicine, Amen Clinics, Costa Mesa, USA

**Keywords:** brain perfusion single-photon emission computed tomography (spect), case report, cognitive slowing, cognitive symptoms, continuous performance test, functional neuroimaging, neuropsychiatric symptoms, obstructive sleep apnea, relative tracer distribution, short-term memory

## Abstract

Persistent cognitive symptoms in older adults may occur in the setting of multiple overlapping neurologic, vascular, sleep-related, psychiatric, and medical factors. We report the case of a 69-year-old man with several years of progressive short-term memory difficulty, cognitive slowing, word-finding difficulty, reduced decisiveness, and collateral concern regarding gait change. The clinical evaluation identified several potentially relevant contributors, including persistent dyslipidemia, inadequately treated obstructive sleep apnea, mild depressive symptoms, and a reported prior cerebrovascular finding. Portions of the outside diagnostic evaluation were unavailable for independent review. Brain perfusion single-photon emission computed tomography (SPECT), obtained as an adjunctive component of the clinical evaluation, demonstrated a nonspecific mixed pattern of relative cortical and subcortical tracer-distribution differences. An individualized management plan was subsequently implemented, focusing on potentially modifiable contributors, sleep optimization, lifestyle measures, and depressive symptoms. At approximately three-month follow-up, the patient’s spouse reported no significant memory improvement but no noticeable decline; subsequent follow-up raised renewed concern regarding progression. Because repeat standardized cognitive testing was not performed and multiple interventions were introduced over time, the longitudinal clinical course could not be objectively quantified or attributed to any individual intervention. This case describes a nonspecific pattern of relative brain perfusion differences in a patient with persistent cognitive symptoms. The relationship between these imaging findings and the patient’s clinical symptoms remains uncertain, and this single observation does not establish a specific neurologic etiology or the diagnostic, prognostic, or treatment-selection utility of brain perfusion SPECT.

## Introduction

Persistent cognitive symptoms are common in older adults and may substantially affect daily functioning, independence, and quality of life. Their evaluation can be challenging because cognitive decline may reflect multiple, potentially overlapping neurodegenerative, cerebrovascular, sleep-related, psychiatric, metabolic, and other medical contributors [1]. Cerebrovascular disease may coexist and interact with other age-related processes affecting cognition [2], while obstructive sleep apnea has been associated with cognitive impairment and cognitive decline in older adults [3]. Depressive symptoms may also coexist with cognitive complaints and further complicate clinical interpretation [4]. Accordingly, determining the potential contributors to cognitive symptoms requires integration of the clinical history, collateral information, cognitive assessment, laboratory evaluation, and other available diagnostic findings.

Neuroimaging represents one component of this broader evaluation. Structural imaging, particularly magnetic resonance imaging (MRI), is commonly used to identify cerebrovascular, structural, and other intracranial abnormalities that may contribute to cognitive impairment [5]. Functional neuroimaging may provide additional information in selected clinical settings but requires interpretation alongside the clinical and conventional diagnostic evaluation. Brain perfusion single-photon emission computed tomography (SPECT) depicts relative regional differences in radiotracer distribution as an indirect representation of cerebral perfusion. Reported perfusion patterns, however, may overlap among cognitive and neurodegenerative disorders, limiting the specificity of individual findings [6].

Brain perfusion SPECT has been investigated as an adjunctive modality in the evaluation of cognitive disorders, although its diagnostic performance varies according to the population and clinical comparison being studied [6]. Structural and functional imaging findings, therefore, should not be interpreted independently of the broader clinical syndrome [5,6]. This distinction is particularly important when several plausible contributors to cognitive symptoms coexist or when portions of the conventional diagnostic evaluation are unavailable for independent review. Accordingly, the clinical significance of regional perfusion differences in an individual patient may remain uncertain even when abnormalities are visually apparent.

We present the case of an older adult with persistent cognitive symptoms who underwent an extensive multidisciplinary evaluation, including brain perfusion SPECT, in the setting of several potential contributing factors and incompletely available outside diagnostic records. The clinical and educational value of this case lies in illustrating a practical approach to evaluating multifactorial cognitive symptoms when conventional diagnostic information is incomplete and adjunctive functional imaging demonstrates nonspecific abnormalities. In particular, the case highlights the importance of distinguishing verified findings from patient-reported outside results, evaluating potentially modifiable contributors such as sleep-disordered breathing, mood symptoms, and vascular risk factors, and interpreting SPECT within the broader clinical context rather than allowing imaging findings to determine diagnosis or treatment. The purpose of this report is therefore to describe the patient’s clinical presentation, available diagnostic evaluation, brain perfusion SPECT findings, multidisciplinary management, and longitudinal clinical course while maintaining appropriate diagnostic uncertainty. As a descriptive case report, this study is not intended to establish a specific neurologic etiology or the diagnostic, prognostic, or treatment-selection utility of brain perfusion SPECT.

## Case presentation

A 69-year-old man presented to Amen Clinics, Costa Mesa, CA, USA, in January 2026 for evaluation of persistent cognitive symptoms that had become progressively more noticeable over approximately two to four years. His principal concern was worsening short-term memory, with relative preservation of long-term memory. He and his spouse described slowed cognitive processing, intermittent word-finding difficulty and pauses or “blanks” during conversation, reduced decisiveness, fatigue, and diminished motivation and engagement in previously enjoyed activities. He also reported reduced appetite and loss of muscle mass. His spouse reported that family and friends had noticed cognitive slowing and a shuffling gait. The symptoms had increasingly affected communication and daily functioning. The available records did not contain a formal assessment of basic or instrumental activities of daily living or sufficiently detailed documentation of driving, medication management, financial management, or occupational functioning; therefore, the degree of functional independence could not be reliably characterized. Family history was notable for short-term memory impairment in his mother at an advanced age.

The available clinical history indicated that he had undergone outside evaluation for his cognitive symptoms during the preceding year. Brain MRI was reportedly interpreted as demonstrating a small stroke involving a posterior brain region, whereas positron emission tomography (PET) imaging was reportedly without significant abnormality. Outside neuropsychological testing was reportedly interpreted as demonstrating mild cognitive decline. According to the patient and his spouse, primary progressive aphasia and Lewy body syndrome had been considered by outside specialists, although neither diagnosis had been established. The original MRI, PET, and neuropsychological reports were unavailable for retrospective review; therefore, the location, extent, and chronicity of the reported cerebrovascular lesion, the type and detailed interpretation of the PET study, and the specific cognitive domains and standardized neuropsychological findings could not be independently characterized. His history also included a remote motorcycle accident at approximately 20 years of age that resulted in a leg fracture; he denied associated head trauma or neurological injury.

Sleep history was notable for heavy snoring, early-morning awakening, daytime fatigue, habitual daytime napping, and previously diagnosed obstructive sleep apnea. Available records documented that a prior sleep study reportedly demonstrated an apnea-hypopnea index of approximately 20 events/hour and that continuous positive airway pressure (CPAP) therapy had been recommended. The original diagnostic sleep-study report was unavailable for retrospective review. He reported difficulty tolerating CPAP and limited adherence. His spouse additionally described occasional yelling, arm movements, and thrashing during sleep. These nocturnal behaviors were characterized by clinical history only; video polysomnography or other objective testing sufficient to characterize a parasomnia was not available.

Neuropsychiatric assessment further characterized his difficulties with short-term memory, cognitive processing, word finding, fatigue, reduced motivation, and anxiety. His anxiety was described primarily in anticipation of medical procedures and evaluations, without reported panic attacks. He reported low energy and diminished interest but denied persistent depressed mood, hopelessness, worthlessness, guilt, or suicidal ideation. Brief cognitive screening with the Self-Administered Gerocognitive Examination (SAGE) yielded a score of 15/22, below the reported normal range [7]. His Patient Health Questionnaire-9 (PHQ-9) score was 7, consistent with mild depressive symptoms [8]. Formal neuropsychological testing had reportedly been performed previously as part of an outside evaluation; however, the original report and domain-level findings were unavailable for retrospective review.

Available routine laboratory data were reviewed for potentially reversible hematologic, metabolic, thyroid, nutritional, renal, and hepatic contributors to his cognitive symptoms (Table 1). Testing from November 2024 demonstrated dyslipidemia, elevated apolipoprotein B, and elevated ferritin. Repeat laboratory testing in December 2025 continued to demonstrate elevated total cholesterol, low-density lipoprotein cholesterol, non-high-density lipoprotein cholesterol, and apolipoprotein B. Broad hematologic, glycemic, thyroid, nutritional, renal, and hepatic testing did not identify another clear biochemical abnormality sufficient to explain the persistent cognitive symptoms. The source laboratory reports confirmed the 2024 hematologic, metabolic, lipid, thyroid, vitamin B12, and ferritin findings and their reported reference ranges. December 2025 testing similarly confirmed persistent lipid and apolipoprotein B abnormalities with otherwise largely unrevealing routine laboratory results.

**Table 1:** Selected routine laboratory evaluation Selected laboratory studies obtained during the metabolic, nutritional, hematologic, and cardiovascular risk evaluation. Persistent dyslipidemia was demonstrated across both testing periods, and ferritin was elevated on earlier testing. Broad hematologic, glycemic, thyroid, nutritional, renal, and hepatic evaluation did not identify a clear reversible biochemical explanation for the persistent cognitive symptoms. Results and reference ranges are presented as reported by the performing laboratories. November 2024 source testing documented the complete blood count, creatinine, alanine aminotransferase, lipid, thyroid, vitamin B12, and ferritin results shown above. December 2025 testing documented creatinine of 90 µmol/L, alanine aminotransferase of 40 U/L, hemoglobin A1c of 5.4%, thyroid-stimulating hormone of 1.58 mIU/L, vitamin B12 of 702 pmol/L, and the listed lipid values. HDL, high-density lipoprotein; LDL, low-density lipoprotein

Laboratory test	November 2024 result	Reference range	December 2025 result	Reference range
White blood cell count	5.1 × 10⁹/L	4.0-11.0 × 10⁹/L	6.8 × 10⁹/L	4.0-11.0 × 10⁹/L
Hemoglobin	165 g/L	135-175 g/L	159 g/L	135-175 g/L
Platelet count	280 × 10⁹/L	140-400 × 10⁹/L	266 × 10⁹/L	140-400 × 10⁹/L
Creatinine	80 µmol/L	50-120 µmol/L	90 µmol/L	50-120 µmol/L
Alanine aminotransferase	40 U/L	<70 U/L	40 U/L	<70 U/L
Hemoglobin A1c	5.60%	4.3-5.9%	5.40%	4.3-5.9%
Thyroid-stimulating hormone	1.38 mIU/L	0.20-6.50 mIU/L	1.58 mIU/L	0.20-6.50 mIU/L
Vitamin B12	417 pmol/L	≥160 pmol/L	702 pmol/L	≥160 pmol/L
Folate	31.7 nmol/L	≥10.0 nmol/L	37.1 nmol/L	≥10.0 nmol/L
Vitamin D, 25-hydroxy	Not available	—	87 nmol/L	50-200 nmol/L
Homocysteine	Not available	—	9.1 µmol/L	≤20.0 µmol/L
Total cholesterol	6.95 mmol/L	<5.20 mmol/L	6.43 mmol/L	<5.20 mmol/L
LDL cholesterol	5.51 mmol/L	<3.50 mmol/L	4.82 mmol/L	<3.50 mmol/L
HDL cholesterol	0.97 mmol/L	≥1.00 mmol/L	1.12 mmol/L	≥1.00 mmol/L
Non-HDL cholesterol	5.98 mmol/L	<4.20 mmol/L	5.31 mmol/L	<4.20 mmol/L
Apolipoprotein B	1.55 g/L	<1.05 g/L	1.35 g/L	<1.05 g/L
Ferritin	653 µg/L	30-500 µg/L	Not available	—

An integrative medicine evaluation further assessed potential metabolic, nutritional, environmental, and infectious contributors. The clinical history included prior exploratory mycotoxin testing reportedly positive for aflatoxin M1, ochratoxin A, and mycophenolic acid in the setting of reported residential water damage. Positive Epstein-Barr virus IgG serology was also reported. The original laboratory reports supporting these exploratory findings were unavailable for retrospective review. Their relationship to the patient’s cognitive symptoms remained uncertain, and these findings did not establish a toxic, active infectious, or inflammatory etiology for the cognitive presentation. The previously documented elevation in ferritin prompted a recommendation for additional hematologic evaluation.

Following the clinical and laboratory evaluation, brain perfusion SPECT was obtained at Amen Clinics in January 2026 as an adjunctive functional imaging study. The clinical SPECT report documented use of technetium-99m hexamethylpropyleneamine oxime (99mTc-HMPAO). Resting and concentration studies were obtained on separate days.

During the concentration study, the patient performed the Conners Continuous Performance Test (CPT), a computerized sustained-attention task requiring responses to target stimuli over time [9]. The CPT served as a standardized sustained-attention condition during the concentration study rather than as a standalone diagnostic assessment of the patient’s cognitive symptoms. According to the clinical SPECT report, radiotracer was administered during CPT performance. This permitted descriptive comparison of the relative regional tracer-distribution pattern during sustained attentional demand with that observed under resting conditions. The paired studies were interpreted descriptively and were not considered quantitative measurements of cerebral activation or absolute cerebral blood flow.

According to the clinical SPECT report, tomographic imaging was performed approximately 30-60 minutes after radiotracer administration using high-resolution gamma-camera imaging with fan-beam collimation. The report described acquisition using a 128 × 128 matrix with 120 projections obtained at 3° intervals over 360°, followed by low-pass prefiltering and linear attenuation correction. Images were reconstructed and reviewed in coronal, sagittal, and transaxial planes and as three-dimensional surface renderings. The patient-specific administered radiotracer activity, exact imaging system, and complete acquisition and reconstruction parameters were not available for retrospective verification; therefore, technical details are presented only as documented in the clinical SPECT report.

The reported regional increases and decreases were based on the qualitative descriptions documented in the clinical SPECT interpretation rather than on validated quantitative regional analysis or a documented normative database comparison available for retrospective review. The resting study was described as demonstrating relatively decreased tracer activity involving the bilateral temporal lobes, including the regions of the bilateral Sylvian fissures, medial dorsal prefrontal cortex, medial parietal cortex, bilateral parietal and occipital regions, prefrontal poles, bilateral inferior orbitofrontal cortex, posterior cingulate region, and cerebellum. A moderate relative decrease was additionally described in the left frontoparietal region, with relative sparing of the remainder of the motor strip. Mild scalloping of the surface-rendered contour was present. These cortical surface findings are shown in Figure 1. The clinical interpretation additionally described relatively increased tracer activity in the left basal ganglia and right thalamus at rest.

**Figure 1:**
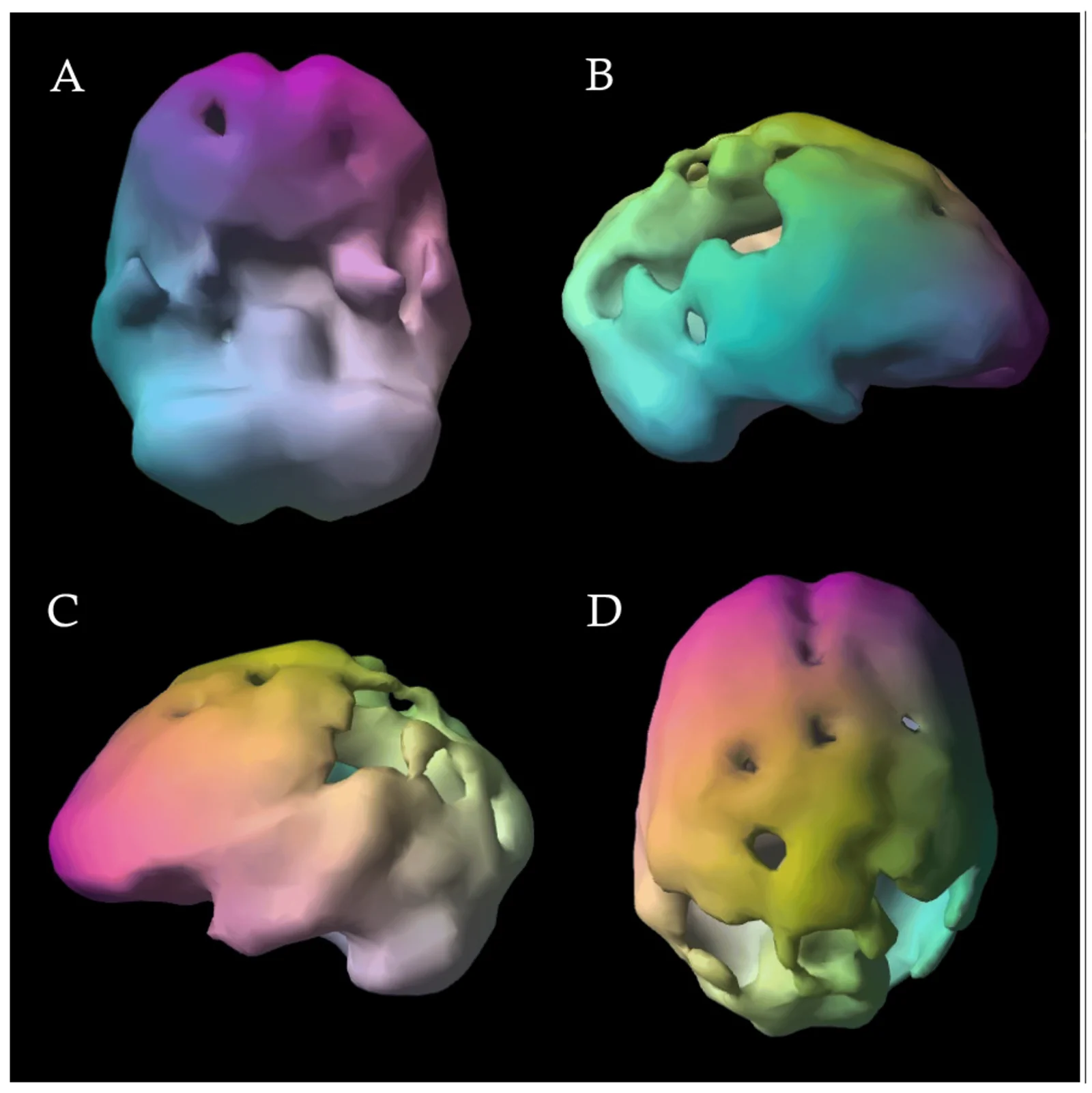
Resting surface-rendered brain perfusion SPECT images Resting surface-rendered brain perfusion SPECT images demonstrating the relative regional distribution of tracer activity. Warmer colors correspond to relatively greater displayed tracer activity, whereas cooler colors correspond to relatively lower displayed tracer activity within the reconstructed brain surface. (A) Inferior (underside) view; (B) Right lateral view; (C) Left lateral view; (D) Superior (top-down) view. The resting study was described as demonstrating relatively decreased tracer activity involving the bilateral temporal lobes, including the regions of the bilateral Sylvian fissures, medial dorsal prefrontal cortex, medial parietal cortex, bilateral parietal and occipital regions, prefrontal poles, bilateral inferior orbitofrontal cortex, posterior cingulate region, and cerebellum. A moderate relative decrease was additionally described in the left frontoparietal region, with relative sparing of the remainder of the motor strip. Mild scalloping of the surface rendering was present. The color and surface-rendered findings represent qualitative differences in displayed relative tracer intensity corresponding to the clinical SPECT interpretation. A numerical color scale, quantitative threshold, reference distribution, and documentation establishing whether the rendering used standardized or patient-specific scaling were unavailable for retrospective review. Accordingly, the color differences and surface contour findings should not be interpreted as quantitative measurements of regional perfusion or absolute cerebral blood flow. These images are presented for descriptive and illustrative purposes and are not independently diagnostic. SPECT, single-photon emission computed tomography

The concentration study was described as demonstrating persistent relatively decreased tracer activity involving the bilateral temporal, medial prefrontal, medial parietal, parietal, occipital, prefrontal pole, inferior orbitofrontal, posterior cingulate, and internal cerebellar regions. According to the qualitative interpretation documented in the original clinical SPECT report, several regional decreases, including those involving the medial parietal, temporal, medial prefrontal, prefrontal pole, inferior orbitofrontal, and left occipital regions, were described as more pronounced during the resting condition; this comparison was not based on validated quantitative between-session analysis. The corresponding cortical surface findings during sustained attentional demand are shown in Figure 2. The clinical interpretation additionally described relatively increased tracer activity in the bilateral basal ganglia and left greater than right thalamus during concentration. These observations represented descriptive differences in relative regional tracer distribution and were interpreted in conjunction with the overall clinical evaluation rather than as independently diagnostic findings.

**Figure 2:**
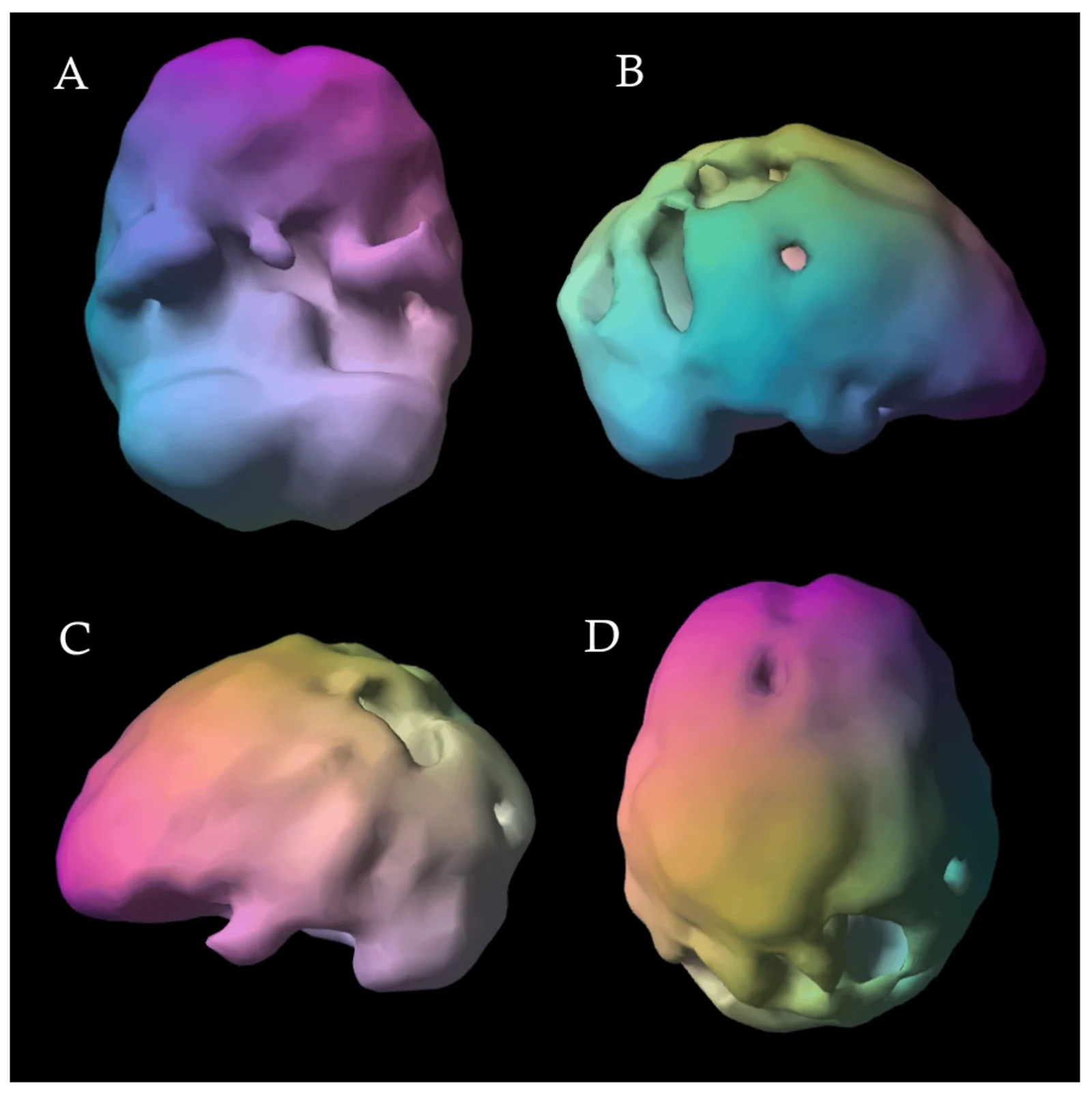
Concentration surface-rendered brain perfusion SPECT images Concentration surface-rendered brain perfusion SPECT images obtained during the Conners CPT, demonstrating relative regional tracer distribution during sustained attentional demand. Warmer colors correspond to relatively greater tracer activity, whereas cooler colors correspond to relatively lower tracer activity. (A) Inferior view; (B) Right lateral view; (C) Left lateral view; (D) Superior view. The concentration study was described as demonstrating persistent relatively decreased tracer activity involving the bilateral temporal lobes, medial dorsal prefrontal cortex, medial parietal cortex, bilateral parietal and occipital regions, prefrontal poles, bilateral inferior orbitofrontal cortex, posterior cingulate region, and cerebellar regions. According to the qualitative interpretation in the clinical SPECT report, several regional decreases, including the medial parietal, temporal, medial prefrontal, prefrontal pole, inferior orbitofrontal, and left occipital regions, were described as more pronounced during rest; this comparison was not based on validated quantitative between-session analysis. Mild scalloping was present in both studies. The color and surface findings represent qualitative differences in relative tracer intensity based on the clinical SPECT interpretation. A numerical color scale, quantitative threshold, reference distribution, and documentation establishing standardized or patient-specific scaling were unavailable for retrospective review. These findings should not be interpreted as quantitative measurements of regional perfusion, cerebral activation, or absolute cerebral blood flow. Rest-concentration differences are presented descriptively, and the images are not independently diagnostic. CPT, Continuous Performance Test; SPECT, single-photon emission computed tomography

Following completion of the initial clinical evaluation, an individualized multimodal management plan was implemented. No psychotropic medication was initially prescribed. Management included dietary modification, regular physical exercise, meditation and stress-management practices, sleep optimization, and continued integrative medical care. Given the history of obstructive sleep apnea and poor CPAP tolerance, adherence to positive airway pressure therapy was encouraged, and otolaryngology evaluation was recommended. Because these interventions were implemented concurrently and were not evaluated individually, no treatment effect could be attributed to any specific component of the management plan.

At follow-up in April 2026, his spouse reported no significant improvement in memory but also no noticeable further decline. The patient described his mood as positive and reported generally feeling well while continuing approximately 20 minutes of daily meditation, regular exercise, dietary modification, and nutritional supplementation. Sleep remained problematic because of poor CPAP tolerance. His spouse continued to report some emotional reactivity. Mental status examination at this follow-up demonstrated neat appearance, normal speech, euthymic mood with congruent affect, normoactive behavior, linear thought process, and good insight and judgment, without suicidal or homicidal ideation or psychotic thought content. Repeat formal cognitive or neuropsychological testing was not performed; therefore, the interval cognitive course was characterized by collateral-reported short-term stability rather than objectively measured improvement.

At a later follow-up, continued concern regarding progression of the cognitive symptoms prompted coordination between the treating clinician and the patient’s family physician. The patient and his family had historically preferred nonpharmacologic and integrative approaches but had become more open to pharmacologic treatment. A geriatric evaluation reportedly included discussion of donepezil and lecanemab; neither treatment was initiated. The original geriatric evaluation was unavailable for retrospective review, and no disease-specific biomarker results were available in the records reviewed for this report. Accordingly, these treatment discussions were considered part of the evolving clinical history rather than evidence establishing a specific neurodegenerative diagnosis.

A subsequent trial of bupropion 150 mg was initiated to address depressive symptoms, reduced motivation, and diminished drive but was discontinued because of mild adverse effects. Following discussion with the family physician, bupropion was restarted at 75 mg with plans for gradual titration as tolerated. During a subsequent telephone check-in, the patient reported variable concentration and conversational ability, mild balance changes, and nighttime dry mouth. He reported subjective improvement in sleep after self-initiated mouth taping; this was not considered a substitute for treatment of obstructive sleep apnea, and positive airway pressure therapy remained difficult to tolerate. These interval changes were patient-reported and were not accompanied by repeat standardized cognitive testing. No acute safety concerns, psychotic symptoms, or manic symptoms were reported.

The documented clinical course, available diagnostic assessments, interventions, and longitudinal follow-up are summarized in Table 2.

**Table 2:** Clinical timeline, diagnostic evaluation, interventions, and follow-up This table summarizes the documented clinical course, available diagnostic assessments, management, and longitudinal follow-up. The original MRI, PET, neuropsychological, polysomnographic, and geriatric evaluation reports were unavailable for retrospective review; corresponding findings are therefore presented as reported clinical history rather than independently verified results. Exploratory environmental and infectious testing was also incompletely documented. Follow-up cognitive changes were patient- or collateral-reported because repeat formal cognitive or neuropsychological testing was not performed. Brain perfusion SPECT findings were interpreted descriptively and were not independently diagnostic. AHI, apnea-hypopnea index; CPAP, continuous positive airway pressure; EBV, Epstein-Barr virus; MRI, magnetic resonance imaging; PET, positron emission tomography; PHQ-9, Patient Health Questionnaire-9; SAGE, Self-Administered Gerocognitive Examination; SPECT, single-photon emission computed tomography

Clinical period	Clinical presentation	Evaluation and key findings	Management and outcome
Several years before index evaluation	Progressive short-term memory difficulty, cognitive slowing, word-finding difficulty and conversational pauses, fatigue, reduced decisiveness, and reduced motivation	Symptoms characterized through patient and collateral history	Progressive symptoms prompted outside and subsequent specialty evaluation
2025 outside evaluation	Persistent cognitive symptoms	MRI reportedly demonstrated a small posterior stroke; PET reportedly showed no significant abnormality; neuropsychological testing reportedly showed mild cognitive decline. Primary progressive aphasia and Lewy body syndrome were reportedly considered but not established. Original reports were unavailable for review	Continued diagnostic evaluation
Late 2025 integrative evaluation	Persistent cognitive symptoms, fatigue, poor appetite, and sleep disturbance	Laboratory evaluation demonstrated dyslipidemia and earlier elevated ferritin without another clear reversible biochemical explanation. Exploratory mycotoxin and EBV findings were reported, but original reports were unavailable	Nutritional, lifestyle, sleep, and integrative interventions were recommended
January 2026: Index clinical evaluation	Persistent short-term memory difficulty, cognitive slowing, word-finding difficulty, fatigue, reduced motivation, and anxiety	Available outside diagnostic documentation remained incomplete. Resting and concentration brain perfusion SPECT demonstrated nonspecific relative regional tracer-distribution differences	Individualized multimodal management was initiated; no psychotropic medication was initially prescribed
Sleep assessment	Heavy snoring, early-morning awakening, daytime fatigue, habitual napping, poor CPAP tolerance, and occasional reported yelling, arm movements, and thrashing during sleep	OSA with reported AHI of approximately 20 events/hour; original diagnostic sleep-study report unavailable. No objective assessment sufficient to characterize a parasomnia was available	CPAP adherence was encouraged; otolaryngology evaluation and sleep optimization were recommended
Neuropsychiatric assessment	Short-term memory difficulty, cognitive processing and word-finding problems, fatigue, reduced motivation, and anxiety	SAGE 15/22; PHQ-9 7. Outside formal neuropsychological testing was reportedly performed, but the original report and domain-level findings were unavailable	Findings were incorporated into the broader clinical evaluation
April 2026 follow-up	Persistent cognitive symptoms	Spouse reported no significant memory improvement but no noticeable further decline. No repeat standardized cognitive assessment was performed	Continued lifestyle, integrative, and sleep-focused management
Later follow-up	Continued concern regarding cognitive progression and reduced motivation	Geriatric evaluation reportedly included discussion of donepezil and lecanemab; neither was initiated. The original geriatric evaluation was unavailable, and no disease-specific biomarker results were available in the records reviewed for this report	Bupropion 150 mg trial was discontinued because of mild adverse effects and was subsequently restarted at 75 mg after coordination with the family physician
Subsequent telephone check-in	Variable concentration and conversational ability, mild balance changes, and nighttime dry mouth	Interval changes were patient-reported; positive airway pressure tolerance remained poor; no repeat standardized cognitive testing was performed	Continued lower-dose bupropion with planned gradual titration

## Discussion

Persistent cognitive symptoms in older adults may occur in the setting of multiple overlapping neurologic, vascular, sleep-related, psychiatric, and medical factors [1]. In the present case, progressive short-term memory difficulty, cognitive slowing, word-finding difficulty, and reduced decisiveness were accompanied by collateral concern regarding gait change and an abnormal brief cognitive screening result. Several potentially relevant contributors were also present, including persistent dyslipidemia, inadequately treated obstructive sleep apnea, mild depressive symptoms, and a reported prior cerebrovascular finding. Although these features support clinically meaningful cognitive dysfunction, they do not establish a single underlying etiology. Accordingly, the clinical presentation is best interpreted as a persistent cognitive syndrome occurring in the setting of several plausible contributing factors.

Several of these potential contributors warrant particular consideration. Cerebrovascular disease and vascular risk factors may coexist with other age-related processes affecting cognition [2]. In this patient, persistent dyslipidemia and the reported prior cerebrovascular finding increased the relevance of possible vascular contributions, although the original magnetic resonance imaging report was unavailable to characterize its location, extent, or chronicity. Inadequately treated obstructive sleep apnea represented another potentially modifiable factor [3]. Randomized-trial evidence suggests that CPAP may improve sleepiness and produce modest improvements in selected neurocognitive outcomes among older adults with obstructive sleep apnea, although the quality and consistency of the evidence remain limited [10]. Mild depressive symptoms, reduced motivation, and decreased drive may likewise have influenced the clinical presentation [4]. However, the available data do not permit any of these factors, individually or collectively, to be identified as the primary cause of the patient’s progressive cognitive symptoms.

Etiologic interpretation was also limited by incomplete access to prior diagnostic studies. The patient was reported to have had a small posterior cerebrovascular abnormality on magnetic resonance imaging, no significant abnormality on positron emission tomography, and mild cognitive decline on formal neuropsychological testing, while outside clinicians had reportedly considered additional neurodegenerative diagnoses. However, the original imaging, neuropsychological, and geriatric reports were unavailable for independent review. The location and clinical significance of the reported cerebrovascular finding, the type and interpretation of the PET study, and the pattern and severity of neuropsychological impairment therefore could not be independently characterized. Because structural imaging and detailed cognitive assessment are important components of the evaluation of persistent cognitive impairment [1,5], these reported outside findings and diagnostic impressions should be regarded as elements of the clinical history rather than independently established diagnoses.

Management was directed toward potentially modifiable contributors and functional symptoms rather than toward a presumed single cause of cognitive decline. At the initial evaluation, no psychotropic medication was initiated, and management emphasized efforts to optimize treatment of obstructive sleep apnea and sleep quality, together with regular physical activity, dietary modification, meditation and stress-reduction strategies, nutritional support, and continued clinical monitoring. Positive airway pressure remained poorly tolerated, and further efforts to address sleep-disordered breathing, including otolaryngologic evaluation, were encouraged. Pharmacologic treatment was introduced later in the clinical course for depressive symptoms, reduced motivation, and decreased drive. Bupropion was selected to target these symptoms rather than as a treatment for the patient’s cognitive impairment or SPECT findings. An initial 150-mg trial was discontinued because of mild adverse effects, after which bupropion 75 mg was reintroduced with a slower titration strategy. Donepezil and lecanemab had reportedly been discussed during an outside geriatric evaluation, but neither was initiated, and no disease-specific biomarker results were available in the records reviewed for this report. These treatment discussions, therefore, did not establish Alzheimer's disease or another specific neurodegenerative diagnosis.

Longitudinal follow-up demonstrated a mixed clinical course rather than a clear treatment response. At approximately three-month follow-up, the patient’s spouse reported no significant improvement in memory but also no noticeable decline. He remained engaged in meditation, exercise, dietary modification, and efforts to improve sleep, although positive airway pressure continued to be difficult to tolerate. Subsequent follow-up raised renewed concern regarding progression, with variable concentration and conversational ability and mild balance changes later reported. Because follow-up cognitive changes were assessed primarily through patient and collateral report without repeat standardized cognitive or formal neuropsychological testing, the magnitude and trajectory of cognitive change could not be objectively quantified. Moreover, several lifestyle, sleep-related, supportive, and pharmacologic interventions were introduced over time. Periods of relative stability or subsequent concern for progression, therefore, cannot be attributed to bupropion, sleep-related interventions, other components of the management plan, or any presumed underlying disease process.

The reported exploratory environmental and infectious findings also require cautious interpretation. The patient previously reported positive mycotoxin testing and possible environmental mold exposure; however, the original source reports were unavailable for independent review, and current clinical guidance does not support blood or urine mycotoxin testing as a validated routine method for diagnosing illness attributed to indoor mold exposure [11]. The available historical Epstein-Barr virus serologic information was likewise insufficient to establish an active infectious contribution to the cognitive presentation. These findings were therefore considered exploratory rather than etiologic. More broadly, the available hematologic, metabolic, thyroid, nutritional, and inflammatory evaluation did not identify a clear reversible biochemical explanation for the cognitive symptoms. Persistent dyslipidemia remained clinically relevant, whereas the previously elevated ferritin was nonspecific and was not considered evidence of a particular inflammatory, infectious, or environmental etiology.

Brain perfusion SPECT provided additional descriptive imaging information but did not resolve the etiologic uncertainty. The clinical interpretation described a mixed pattern of relative cortical and subcortical tracer-distribution differences involving multiple brain regions. Published evidence demonstrates overlap in perfusion patterns across cognitive disorders and imperfect diagnostic performance of SPECT [6]. Brain perfusion SPECT using technetium-99m-labeled radiopharmaceuticals represents relative regional tracer distribution related to perfusion rather than a direct quantitative measurement of absolute cerebral blood flow [12]. Accordingly, the observed findings should be regarded as nonspecific adjunctive observations within the broader clinical evaluation and not as evidence establishing a particular neurodegenerative, cerebrovascular, traumatic, or other neurologic diagnosis.

Several limitations should be considered. This report is based on a retrospective clinical record, and the original outside MRI, PET, formal neuropsychological testing, polysomnography, and geriatric evaluation reports were unavailable for review. The reported cerebrovascular finding, therefore, could not be independently characterized; the type and interpretation of the prior positron emission tomography study were unknown, and the detailed neuropsychological profile and reported severity of obstructive sleep apnea could not be independently verified. Repeat standardized cognitive or formal neuropsychological testing was not available to objectively quantify longitudinal change. For the SPECT studies, the patient-specific administered radiotracer activity, exact imaging system, and complete acquisition and reconstruction parameters were unavailable for retrospective verification. Validated quantitative outputs, documented normative comparator data, and formal inter-reader assessment were also unavailable for retrospective inclusion. Patient-preparation conditions were also incompletely documented, and because the resting and concentration studies were acquired on separate days, between-session physiologic variability may have contributed to apparent differences between the studies. Age, vascular risk, sleep-disordered breathing, psychiatric symptoms, medications and supplements, the reported prior cerebrovascular finding, and other unrecognized factors may have influenced the clinical or imaging findings. Finally, because multiple interventions were introduced during the clinical course, subsequent stability or progression cannot be attributed to any individual treatment. As a single descriptive case, these observations do not establish a specific pathophysiologic mechanism or the diagnostic, prognostic, or treatment-selection utility of brain perfusion SPECT.

## Conclusions

Persistent cognitive symptoms in older adults remain diagnostically challenging, particularly when several plausible contributing factors coexist, and portions of the conventional diagnostic evaluation are unavailable for independent review. This case illustrates the importance of distinguishing verified findings from reported outside results, evaluating potentially modifiable contributors, and integrating adjunctive functional imaging within the broader clinical context rather than allowing imaging findings to determine diagnosis or treatment. Brain perfusion SPECT demonstrated a nonspecific mixed pattern of relative cortical and subcortical tracer-distribution differences, but the relationship between these findings and the patient’s cognitive symptoms remained uncertain. This single case does not establish a specific neurologic etiology or the diagnostic, prognostic, or treatment-selection utility of brain perfusion SPECT. Prospective studies incorporating standardized clinical phenotyping, objective longitudinal cognitive assessment, conventional diagnostic characterization, quantitative neuroimaging methodologies, and longitudinal follow-up are needed before the potential clinical role of brain perfusion SPECT in patients with persistent cognitive symptoms can be defined.
